# A Rare Event

**Published:** 1890-02-22

**Authors:** 


### The Hospital

A Weekly Journal of

**Science, Medicine, Nursing, and Philanthropy.**

*For Hospitals, Asylums, Medical Practitioners, Students, Nurses, Families, Parishes, Congregations, and the Charitable Public.*

Vol. VII.—No. 178.] [February 22, 1890.

### A Rare Event.

Doctors receive a considerable amount of abuse at the hands of the public, a little of which they may deserve, though a good deal of it is purely gratuitous. It is very seldom that a number of physicians and surgeons are marked out in a body for special public honour and distinction. All the more, therefore, when such an event does take place are we disposed to give it prominent and permanent record. To the town of Dewsbury belongs the peculiar merit of having discovered the excellent worth of all the members of its hospital staff, and of having set apart a special occasion on which all classes of the people could unite to do them honour. A meeting was held at the hospital a few days ago which, as the president said, was quite unique in the history of the institution; and, he added, probably unique in the history of hospitals. At that meeting each member of the medical and surgical staff, from the senior physician to the dental surgeon, was asked to accept a valuable gift as a public token of the estimation in which his work and character were held by all ranks in the town. Without effort of any kind a sum of no less than two hundred pounds was raised in a very short time for the purposes of this remarkable testimonial. Of that amount the members of the Heckmondwike Co-operative Society unanimously voted twenty-five pounds. The remainder was given in various sums down even to the few pence of the widow and the workman.

Whenever anything happens that never happened before, even if it be nothing more than the advent of a Barnum's Show, or the introduction of a new beetle to a village museum, it is always worth a passing glance and a moment's consideration. In medical circles especially anything of a novel character demands a little thought. For nothing, perhaps, is so obnoxious to a genuine doctor as that which is really new. Ordinarily he is like a carriage horse in the London streets freshly imported from the Yorkshire Wolds. If but so much as a strange piece of paper be lying in the road he starts and trembles, and in all probability bolts. But this innovation at Dewsbury is of a nature to soothe the most sensitive doctor, and to reassure the timidest. Even a physician or surgeon can publicly receive the gift of a gold watch or a handsome clock, if only other physicians and surgeons accept similar gifts at the same time. Were one doctor alone to be asked to accept such a testimonial on a public occasion he would be more than usually valiant if he acceded to the request. For might not some amiable *confrère* write to the *Bistoury*, and charge him with self-advertisement?

But to return! What auspicious star has presided over the nativity and growth of the Dewsbury Hospital so happily as to produce such genuine accord between the different members of the medical staff; between the staff as a body and the committee of management; between the hospital itself and all classes of the public, as to give rise to such a spontaneous and generous recognition of professional services as we have recorded? It is not always that such internal harmony prevails at hospitals as makes the whole community recognise and admire their work. But why is it not so? And why does this event at Dewsbury stand out as the only one of its kind in the hospital story? By way of possible answer to these questions, a glance at the *personnel* of the medical staff may give some help.

It is an extremely significant fact, at any rate from the point of view of the initiated, that every individual member of the Dewsbury Hospital staff is what is known as a "general practitioner." We believe there never has been at Dewsbury either a pure consulting physician or a pure consulting surgeon. There is no pure consultant nearer than the town of Leeds, if indeed there be one there. Still more remarkable is it that of the four members of the surgical staff only one possesses the qualification of F.R.C.S., which qualification alone would fit him to occupy an honorary position in most of the other hospitals in London and the country. With this solitary exception all the surgeons of the hospital, four or five in number, hold the diploma of M.R.C.S. only. With regard to the physicians, not one is a Fellow or even a Member of any one of the Royal Colleges. Two of them are graduates of British Universities and hold no other medical qualification at all. Yet the chairman of the committee affirms, and we fully believe it, that no hospital in Great Britain has produced better results than those accomplished by the staff of the Dewsbury Infirmary. When to this it is added that those provincial physicians and surgeons have proved themselves to be such experienced men of the world, and so uniformly courteous to the managers and kind to the patients as to have secured the universal approval of all classes in Dewsbury and the neighbourhood, a case is made out for the general practitioner which is absolutely unassailable. The truth is that, thanks to recent regulations, all fully qualified medical men in Great Britain are now at starting practically on a level as regards general and professional education. Any superior merit that may be possessed by particular individuals must be proved by their actual work in medical and surgical practice.

From the point of view hitherto taken the interest of this unique event chiefly concerns medical men, and especially general practitioners. But all those who take up hospital work in any capacity whatever must see that a state of public feeling such as has been disclosed at Dewsbury is exactly what ought to prevail in every locality where a voluntary hospital is placed. That state of public feeling is clearly the result of the admirable behaviour of the medical and surgical staff. The effect of this upon the finances and popularity of the institution has been such as every reader will already have been able to predict. From the foundation of the hospital a few years ago to the present time the resources furnished by the public have steadily increased, and at this moment the material prosperity of the institution stands at its maximum.

The state of things at Dewsbury, which is one of the most important centres of population in Yorkshire, affords matter for comparison with what is too often the feeling concerning hospitals in London. In the metropolis adverse criticism is frequently the order of the day rather than enthusiastic approval. That criticism comes from two very different quarters —the newspaper press, and the general practitioners of medicine. We do not say that it is always just, or that it is always according to knowledge. On the contrary, it is certain that many newspaper critics undertake to pronounce verdicts on subjects which they know nothing at all about; and it is also true that the general practitioner, harassed, straitened, and made desperate by the pressure of the times, falls foul of the hospitals because they are the most convenient objects on which he can vent his dissatisfaction. But though it is admitted that hospitals receive a liberal measure of unjust abuse, it cannot be denied that they might do much more than they do both to disarm the criticism of the newspapers and to soothe the natural irritation of the general practitioners. It is to the latter point that, prompted by the happy incident at Dewsbury, we desire to direct special attention at the present moment. We ask hospital managers, and the physicians and surgeons of those institutions as well, whether it is not possible to devise some means whereby every worthy general practitioner may become associated directly with one or other of the large general hospitals? There must be—there is—among general practitioners a number of men of quite exceptional merit and culture. Those men are as much superior to the ordinary and undistinguished hospital physician and surgeon as can well be imagined. Why is no attempt ever made to recognize their merit and worth? Not only are those meritorious persons themselves injured by being left in the cold shade of hospital neglect, but the whole profession in the metropolis is deprived of the most powerful and wholesome stimulus to exertion that is known in medicine. But the evil extends further than this; for by the operation of the present system many hospitals are manned by second and third-rate physicians and surgeons, which, if their managers had but courage and capacity, might be served by men of the very highest order of professional merit. This is a subject which is pre-eminently ripe for consideration at the present moment; and we are glad that the Dewsbury incident has enabled us to give a point to our moral which the most sceptical will not be able to evade.

